# CMAT: ClinVar Mapping and Annotation Toolkit

**DOI:** 10.1093/bioadv/vbae018

**Published:** 2024-02-07

**Authors:** April Shen, Marcos Casado Barbero, Baron Koylass, Kirill Tsukanov, Tim Cezard, Thomas M Keane

**Affiliations:** European Molecular Biology Laboratory, European Bioinformatics Institute, Wellcome Genome Campus, Hinxton, Cambridge CB10 1SD, United Kingdom; European Molecular Biology Laboratory, European Bioinformatics Institute, Wellcome Genome Campus, Hinxton, Cambridge CB10 1SD, United Kingdom; European Molecular Biology Laboratory, European Bioinformatics Institute, Wellcome Genome Campus, Hinxton, Cambridge CB10 1SD, United Kingdom; European Molecular Biology Laboratory, European Bioinformatics Institute, Wellcome Genome Campus, Hinxton, Cambridge CB10 1SD, United Kingdom; European Molecular Biology Laboratory, European Bioinformatics Institute, Wellcome Genome Campus, Hinxton, Cambridge CB10 1SD, United Kingdom; European Molecular Biology Laboratory, European Bioinformatics Institute, Wellcome Genome Campus, Hinxton, Cambridge CB10 1SD, United Kingdom

## Abstract

**Summary:**

Semantic ontology mapping of clinical descriptors with disease outcome is essential. ClinVar is a key resource for human variation with known clinical significance. We present CMAT, a software toolkit and curation protocol for accurately enriching ClinVar releases with disease ontology associations and complex functional consequences.

**Availability and implementation:**

The software and ontology mappings can be obtained from: https://github.com/EBIvariation/CMAT.

## 1 Introduction

Semantic mapping of clinical descriptors with disease outcome is essential for systematic usage of clinical data resources. ClinVar is a highly accessed publicly available archive of human genetic variants and their clinical significance, including their association with diseases and other clinical phenotypes (Landrum *et al.* 2020). ClinVar has proved valuable not just as a primary resource but is also integrated with other resources, such as gene and disease databases, to facilitate the discovery and interpretation of novel genetic variants. However, ClinVar data is highly heterogeneous in variant formats, annotation, and disease descriptors which limits its interoperability. Aggregation and integration requires terminology to be harmonized across multiple sources as well as additional information to be brought in to support downstream use cases.

Users of ClinVar include clinical genetics laboratories, human genetic variation resources, and particularly human genetic aggregation platforms (e.g. Ensembl, UCSC genome browsers). They require structured data and uniform annotations for parsing and processing releases from ClinVar, to ensure these resources’ data is kept consistent and up to date over time. Existing software for parsing and exploring ClinVar data tends to focus on visualization and interrogation of the existing data rather than augmenting or transforming it (Henrie *et al.* 2018, Yauy *et al.* 2022).

To this end we have created the ClinVar Mapping and Annotation Toolkit (CMAT). This freely available software toolkit and curation protocol is designed to aid in parsing and enriching ClinVar’s XML releases, by: (i) mapping genes and traits to specific nomenclatures; and (ii) annotating records with additional information, in particular functional consequences of complex variants. CMAT has been in use by the Open Targets Platform (Ochoa et al. 2023), a public resource for drug target identification and prioritization, since 2016. As part of this process, we have generated a rich and up-to-date set of mappings for traits in ClinVar to the Experimental Factor Ontology (EFO) (Malone *et al.* 2010), which are released regularly with the software.

## 2 Implementation

**Figure 1.**
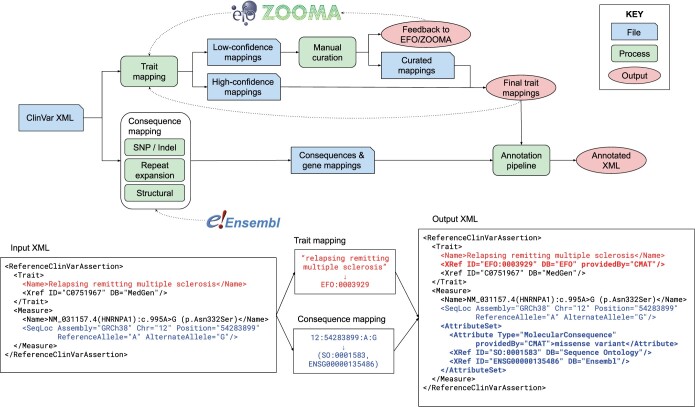
Top: CMAT workflow, including automated and manual processes, intermediate files, and indication of where external APIs are leveraged. Bottom: an example of CMAT’s annotation applied to a ClinVar record, showing how the trait “Relapsing remitting multiple sclerosis” is mapped to EFO:0003929 and that the variant 12:54283899: A:G is a missense mutation (SO:0001583) on the gene ENSG00000135486. Portions of the XML are omitted for clarity.

CMAT consists of three modules: trait mapping protocol, functional consequence pipeline, and the annotation pipeline. The input is the latest full ClinVar XML release (https://ftp.ncbi.nlm.nih.gov/pub/clinvar/xml/). Figure 1 depicts the process along with an example annotation of both trait and variant. The complete protocol including both automated and manual portions is included as part of the CMAT package.

### 2.1 Trait mapping and manual curation

The trait mapping protocol aims to map trait names from ClinVar to a single ontology of the user’s choice. The default ontology and the one with mappings included with the software is EFO, a suitable ontology for aggregation applications as it is both broad in scope and well-aligned with more specialized resources, such as the Mondo Disease Ontology (MONDO) (Vasilevsky *et al.* 2022). In addition, existing collaboration between maintainers of EFO and CMAT allows for a tight feedback loop. Processing by CMAT continuously improves the ontology by requesting terms be added to EFO, either imported from compatible ontologies such as MONDO or newly created based on other information in the ClinVar record, such as MedGen identifiers.

The trait mapping process first attempts to automatically map trait names from ClinVar to terms and synonyms in the chosen ontology. For this purpose, CMAT uses ZOOMA (https://www.ebi.ac.uk/spot/zooma/) and OxO (https://www.ebi.ac.uk/spot/oxo/), tools designed to map text to ontology terms and find cross-references respectively. From these we find high-confidence ontology mappings for each trait. Lower confidence mappings are then presented for curators to manually review and ensure accuracy and currency. Finally, the results of the manual curation are integrated into CMAT’s repository of previous mappings, generated by previous instances of this process and released alongside the software, allowing for their incorporation into future iterations. The process also generates feedback not just for EFO as described above, but also for ZOOMA, which enables other users of that service to utilisz the results of the curation. This feedback process can also be adapted for other ontologies.

### 2.2 Functional consequence and gene mapping

CMAT annotates variants in ClinVar records with genes and functional consequences by annotating Ensembl stable gene and transcript IDs. For relatively simple variants like SNPs and short indels, the pipeline relies on Ensembl’s Variant Effect Predictor (VEP) (McLaren *et al.* 2016) which calculates consequences independently for each variant. Complex events, such as repeat expansion variants, are less frequent but can be of high clinical importance. They are represented in a multitude of ways in ClinVar, particularly HGVS notation, which is able to capture complexities like imprecise locations and variable number of repeats. CMAT processes both the HGVS notation and the variant coordinates where present and separates variants according to their type. Repeat expansion variants are annotated as trinucleotide or short tandem repeat expansions based on the length of the repeating element, and the affected gene is determined based on location. Large insertions and deletions with precise locations are provided to VEP using the appropriate structural variant type indication. This composite approach provides a controllable and extensible framework for expanding to other complex variation types.

### 2.3 Annotation pipeline

The annotation pipeline integrates the information from the previous components and produces the enriched dataset, either in the form of an annotated ClinVar XML file or user-defined text format. The consequence mapping pipeline, annotation pipeline, and the automated portions of the trait mapping protocol are all implemented in portable Nextflow pipelines and utilize reusable Python modules to manage ClinVar records, variants, and traits.

## 3 Results

We ran CMAT (v3.0.2) on the July 2023 full XML release from ClinVar to ascertain its ability to parse and annotate records with high coverage and accuracy. We executed the trait mapping and manual curation protocols to create an up-to-date set of trait mappings to EFO (v3.56.0), followed by the automated functional consequence pipelines leveraging Ensembl 110 to generate the final annotated XML (Fig. 1).

ClinVar routinely annotates the traits it provides to several ontologies such as MONDO, MedGen, Orphanet or OMIM and rarely (0.2%) explicitly annotates terms as being present in EFO. To evaluate our mappings to EFO, we compare them to terms provided by ClinVar that are already present in EFO, even if not explicitly annotated as such. We refer to these as EFO terms provided by ClinVar.

Overall CMAT annotates 98.6% of traits with current EFO terms, compared to the 59.6% of traits with current EFO terms provided by ClinVar. This is attributable not only to our explicit focus on EFO as the target ontology, but also to the fact that the protocol itself includes regular feedback and import of terms into EFO.

We also evaluated concordance between CMAT’s and ClinVar’s ontology terms across their respective ontologies. This is a difficult problem due to the complexity and heterogeneity of the different biomedical ontologies used (Kamdar *et al.* 2017, Xue *et al.* 2019), but overall we estimate the two annotations are equivalent about 98.5% of the time when both are present for a given trait. Details on our methodology can be found in the supplementary material.

CMAT annotates an Ensembl gene ID for 97.0% of variants, which are equivalent to the HGNC gene IDs provided by ClinVar in 99.9% of cases where both are present for a given variant. CMAT also annotates 97.0% of variants with functional consequences, as opposed to 96.4% of variants with consequence annotations from ClinVar. CMAT’s annotations are equivalent to consequences in ClinVar in 91.8% of cases where both are present. What divergences exist we attribute primarily to differing use cases. One situation where this occurs is for repeat expansion variants, for which CMAT uses specific terms to distinguish between trinucleotide repeats and other short tandem repeats, terms that are not used in ClinVar at all. CMAT also contains special processing for complex variants without precise coordinates, a small but clinically important fraction of ClinVar. CMAT currently processes 949 such complex variants, 95.7% of which do not have functional consequence annotations in ClinVar.

Finally, we manually gathered statistics on feedback provided to EFO and ZOOMA through regular CMAT processing. On average we submit 132 terms to EFO per year, both imported and newly created. Additionally 97.6% of trait mappings used in the annotation originate from previous CMAT processing, primarily manual curation, which is incorporated into the ZOOMA service so it can benefit other users as well.

## 4 Discussion

We describe CMAT, a software toolkit and curation protocol for accurately enriching ClinVar releases with disease ontology associations and complex functional consequences. CMAT has been in use since 2016 in providing ClinVar’s variant and phenotype associations to the Open Targets Platform, generating approximately 3 million pieces of evidence from over 97% of ClinVar records and testifying to the efficacy of the system as a whole. In the future, we hope to continue to improve our processes, particularly our manual curation protocol; increase our support of complex variation types such as translocations and imprecise structural variants; support for ClinVar records that associate a trait with multiple variants; haplotype aware variant consequence annotation; and increase the flexibility and extensibility of the software, so it can be more easily adapted to other nomenclatures and annotations.

## Supplementary Material

vbae018_Supplementary_Data
