# Supplementary material for: CMAT: ClinVar Mapping and Annotation Toolkit
Source: Bioinform Adv. 2024 Feb 7;4(1):vbae018. doi: 10.1093/bioadv/vbae018 (PMC10879749; doi:10.1093/bioadv/vbae018)
Supplement: vbae018_Supplementary_Data [file vbae018_supplementary_data.pdf]

Bioinformatics Advances, 2023, 1–1
doi: DOI HERE
Advance Access Publication Date: Day Month Year
Application Note

Software

CMAT: ClinVar Mapping and Annotation Toolkit

April Shen,[1,†] Marcos Casado Barbero,[1,†] Baron Koylass,[1] Kirill Tsukanov,[1] Tim Cezard[1,*] and Thomas M. Keane[1,*]

[1]European Bioinformatics Institute, Wellcome Genome Campus, Hinxton CB10 1SD, UK

[†]Joint first authors.[*]Corresponding authors.

Abstract

Supplementary Material

Evaluation of trait mapping

Trait mapping is restricted to ClinVar records with specific informative traits (i.e. not ones labelled "not provided" or "not specified"), as they are the only ones we can map to EFO terms. For these mapped traits, we wish to compare CMAT's annotations with any EFO terms already present in the original ClinVar record, to evaluate whether CMAT is able to increase coverage of EFO annotations while remaining consistent with existing information.

Several factors complicate the measure of consistency, including deduplication and specificity. First, ontologies do not always consistently reuse or deduplicate terms. This means that terms that overlap in their meaning may not be directly linked by their IRIs, references or mappings, hence creating two different terms that represent the same or a similar concept. Secondly, term annotations can have slightly different specificity, such as "thrombophilia due to protein C deficiency, autosomal recessive" (MONDO:0012860) vs. "hereditary thrombophilia due to congenital protein C deficiency" (MONDO:0019145), without necessarily being invalid. We consider two mappings equivalent if they are identical or annotated as synonyms in EFO. For the evaluation we use the Ontology Lookup Service (OLS, `https://www.ebi.ac.uk/ols4`) to examine these mappings automatically, noting that a fully comprehensive comparison of the annotated terms would require manual effort.

Finally, sometimes traits may be assigned multiple ontology terms by ClinVar; we consider the CMAT mappings equivalent to the set of ClinVar ontology terms if at least one of the CMAT terms is equivalent to at least one of the ClinVar terms. This is justified as both sets of terms are presumably equivalent as they are annotating the same trait. Table 1 provides the results given these definitions.

Evaluation of gene and consequence mapping

For the comparison of ClinVar's and CMAT's genes and functional consequences, we process only records dealing with single variants, rather than other complicated event types (e.g. haplotypes). This is due to the fact that the latter constitutes only a minute percentage of ClinVar records while presenting multiple challenges with regards to sound functional consequence prediction. We map HGNC gene IDs in ClinVar to Ensembl gene IDs using BioMart (Durinck et al., 2009) in order to compare them with our annotations. Functional consequences can be compared directly as both ClinVar and CMAT use Sequence Ontology terms (Eilbeck et al., 2005). Again, we must compare sets of annotations, as variants can impact multiple genes and have multiple molecular consequences. The results are in Table 2.

Table 1. Results for trait mapping. Total records derived from the June 2023 full ClinVar XML release (`https://ftp.ncbi.nlm.nih.gov/pub/clinvar/xml/`). Bear in mind that records may have a variable number of traits.

**Total records**	**2,982,171**	**100%**
Records with informative traits	1,915,650	64.2%
**Total informative traits**	**2,215,121**	**100%**
ClinVar annotation missing	867,080	39.1%
CMAT annotation missing	1,207	0.1%
Both missing	29,011	1.3%
Both present and equivalent	1,298,308	58.6%
Both present and inequivalent	19,515	0.9%

References

S. Durinck et al. Mapping identifiers for the integration of genomic datasets with the R/Bioconductor package biomaRt. *Nature Protocols*, 4:1184–1191, 2009.

K. Eilbeck et al. The Sequence Ontology: a tool for the unification of genome annotations. *Genome Biology*, 6:R44, 2005.

© The Author 2023. Published by Oxford University Press. All rights reserved. For permissions, please e-mail: journals.permissions@oup.com

Table 2. Results for genes and consequences. Total records derived from the June 2023 full ClinVar XML release (`https://ftp.ncbi.nlm.nih.gov/pub/clinvar/xml/`).

**Total records**		**2,982,171**	**100%**
Records with single variants		2,978,840	99.9%
**Total single variants**		**2,978,840**	**100%**
Genes	ClinVar annotation missing	336	0.0%
	CMAT annotation missing	76,240	2.6%
	Both missing	13,395	0.4%
	Both present and equivalent	2,885,889	96.9%
	Both present and inequivalent	2,980	0.1%
Functional consequences	ClinVar annotation missing	19,300	0.6%
	CMAT annotation missing	2,792	0.1%
	Both missing	86,843	2.9%
	Both present and equivalent	2,634,364	88.4%
	Both present and inequivalent	235,541	7.9%
